# Speed Sensorless Control of Linear Ultrasonic Motors Based on Stator Vibration Amplitude Compensation

**DOI:** 10.3390/s20226705

**Published:** 2020-11-23

**Authors:** Yuzhao Yan, Ming Yang, Tianyue Yang, Siwei Ye, Wanlu Jiang

**Affiliations:** 1 Department of Instrument Science and Engineering, Shanghai Jiao Tong University, Shanghai 200240, China; yan0318@sjtu.edu.cn (Y.Y.); thomas-yang@sjtu.edu.cn (T.Y.); yesiwei@sjtu.edu.cn (S.Y.); 2 Hebei Province Key Laboratory of Heavy Machinery Fluid Power Transmission and Control, Yanshan University, Qinhuangdao 066004, China; wljiang@ysu.edu.cn

**Keywords:** linear ultrasonic motor, stator vibration amplitude compensation, speed sensorless control

## Abstract

In some applications of linear ultrasonic motors (LUSMs), not installing speed/position sensors can reduce the size and cost of the system, changes in load will cause fluctuations in the speed of the LUSM. To eliminate the influence of load changes on speed, a speed sensorless control scheme based on stator vibration amplitude compensation (SSCBVC) is proposed. This scheme is implemented under the framework of the stator vibration amplitude-based speed control (VBSC) and frequency tracking. Based on the stator vibration amplitude-speed and the output force-speed curves of the LUSM, the relationship between the load changes and stator vibration amplitude (SVA) to be compensated is established, realizing a speed sensorless control of the LUSM under variable load conditions. The experimental results show that the maximum fluctuation of the speed is about 2.2% when the output force changes from 0 to 6 N with SSCBVC. This scheme can effectively reduce the influence of load changes on the speed of the LUSM without using speed/position sensors.

## 1. Introduction

Linear ultrasonic motors (LUSMs) have the advantages of high control accuracy, simple structure, and quick response [1], and have been widely used in metrology, aerospace, and other fields [2,3,4]. According to the mechanical characteristics of the LUSM, changes in load will cause large fluctuations in the speed of the LUSM [5,6]. In some applications, LUSMs need to operate under different loads, therefore the impact of load changes cannot be ignored in speed control.

To control the speed of the ultrasonic motor, different control algorithms, such as proportional-integral-derivative (PID) control [7], PID control with dead zone compensation [8], and auto regressive (AR) model control [9] are proposed. These methods use the speed/position sensor to feedback the speed of the ultrasonic motor to form a closed-loop control. In the case of load changes, the speed of the ultrasonic motor is still stable. Magnetic grid displacement sensors, laser displacement sensors, and grating displacement sensors are commonly used to measure the speed/position of the ultrasonic motor [10,11,12]. The magnetic gate displacement sensor is easy to install and has a fast response speed, but its measurement accuracy is poorly compared with the optical sensor and it requires high machining accuracy during design and production [10]. The laser displacement sensor has high measurement efficiency, high measurement accuracy, and wide measurement range, but it is bulky and requires a large installation space [11]. The grating displacement sensor not only has high measurement accuracy and wide measurement range, but also has the advantages of simple and compact structure and small volume, it is more suitable for some applications where the system volume is limited, but it is sensitive to dust and dirt, so it has higher requirements for the measurement environment [12]. Furthermore, the installation of the sensors will increase costs [13]. Many speed/position sensorless control methods for synchronous motors with good performance have been proposed [14,15]. These methods are based on the electromagnetic motor model, but the motor model used for performance estimation of the ultrasonic motor is very complicated due to the nonlinear characteristics of the ultrasonic motor, so the speed sensorless control method based on the motor model is not practical for the ultrasonic motor [16]. To achieve speed sensorless control of the ultrasonic motor, some algorithms based on neural networks, such as recurrent fuzzy neural network [17] and genetic neural network [18], have good performance. Because the neural network has good nonlinear identification ability, it can effectively predict the relationship between the input variables and speed of the ultrasonic motor. Therefore, the speed of the ultrasonic motor can be estimated based on these input variables without a speed/position sensor. However, the neural network is relatively complicated and requires a lot of calculation time [13]. Senjyu et al. [16] proposed a method for estimating the rotor position of a rotary ultrasonic motor based on the input voltage, and the algorithm of this method is relatively simple. This method uses the characteristic of the phase of the rotor position is in good agreement with the phase of the input voltage. However, for LUSMs, the slider position change is not associated with the input voltage, so this method is not suitable for LUSMs. Fang et al. [19] found that the transformer tap voltage of the transformer ratio arm bridge is linear to the rotor speed of the rotary ultrasonic motor. Therefore, the rotor speed can be estimated according to the transformer tap voltage. This method is based on the linear relationship between the SVA and the rotor speed of the rotary ultrasonic motor. This relationship also exists between the SVA and the slider speed of the LUSM [20]. However, this method also requires that the load of the LUSM is constant, and changes in load will cause fluctuations in the slider speed, resulting in a non-linear relationship between the SVA and slider speed. Therefore, using a simple algorithm to achieve speed sensorless control of LUSMs under variable load conditions remains a challenge.

In some applications of LUSMs, not installing speed/position sensors can reduce the size and cost of the system. To solve the problem of the speed fluctuation caused by the load change, a speed sensorless control scheme based on stator vibration amplitude compensation (SSCBVC) is proposed. The algorithm of this scheme is simple and easy to implement in microcontrollers. Firstly, the relationship between the stator vibration amplitude (SVA) and speed, the load and speed of the LUSM, the principle of the stator vibration amplitude-based speed control (VBSC) and the frequency tracking are analyzed. Secondly, the specific implementation scheme for the SSCBVC is introduced. Finally, the feasibility of the SSCBVC is verified by comparing the speed fluctuations before and after compensation when the load changes.

## 2. Theoretical Analysis

### 2.1. Relationship Between the Stator Vibration Amplitude and Speed, the Load and Speed of the Linear Ultrasonic Motor

**Figure 1:**
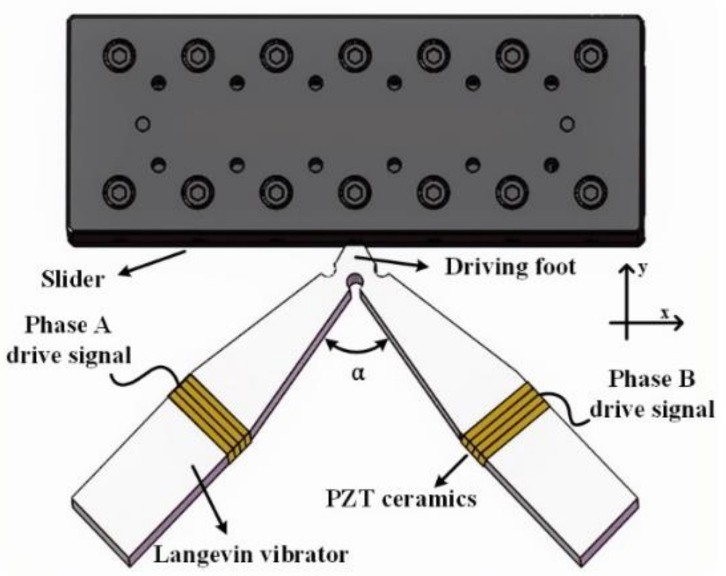
Structure of the V-type linear ultrasonic motor.

The LUSM is mainly composed of a stator and a slider. For this experiment, a V-shaped LUSM is used and the structure of it is shown in Figure 1. The stator is composed of two Langevin vibrators, which are distributed in a V shape [21]. Two-phase specific drive signals are applied to the piezoelectric (PZT) ceramics to excite the symmetric mode and anti-symmetric mode of the stator, so that the vibration trajectory of the driving foot is an ellipse. Then the driving foot is in contact with the slider and the driving foot drives the slider to move linearly under the effect of friction. The speed of the slider is called the speed of the LUSM. The motion trajectory equation of the driving foot is shown in Equation (1):

(1) $${\left(\frac{a_{x}}{A_{x}}\right)}^{2}+{\left(\frac{a_{y}}{A_{y}}\right)}^{2}=1,$$

where $a_{x}$ and $a_{y}$ are the displacement response of the driving foot in the horizontal and vertical direction, respectively. $A_{x}$ and $A_{y}$ are the vibration amplitude of the driving foot in the horizontal and vertical directions, respectively, called the horizontal SVA and the vertical SVA. Generally, the angle α between the two Langevin vibrators is 90°, the signal amplitudes of the two-phase drive signals are equal and the phase difference between the two-phase drive signals is 90°, so $A_{x}=A_{y}=A$, which means the horizontal SVA is equal to the vertical SVA, therefore, collectively call them SVA, and the motion trajectory of the drive foot is a circle.

Li et al. [20] did a research on the relationship between the SVA and speed of the LUSM, and the research showed that under constant load, when the SVA changes within a certain range, it is linear with the speed of the LUSM. Therefore, controlling the speed based on the SVA, i.e., VBSC, can improve the control performance. The relationship between the SVA and speed $v_{1}$ can be expressed by the following equation:

(2) $$v_{1}=k_{1}A+b_{1},$$

where A is the SVA of the driving foot, $k_{1}$ and $b_{1}$ represent the slope and intercept, respectively. Because the speed becomes faster as the SVA increases, $k_{1}\;$> 0.

Shi et al. [22] did a research on the relationship between the load and speed of the LUSM. The results show that under the condition of constant SVA, as the load increases within a certain range, the speed decreases approximately linearly. In the region where load and speed are approximately linear, in order to reduce the complexity of the algorithm, the relationship between the load and speed $v_{2}$ under constant SVA can be assumed to be inversely proportional as below:

(3) $$v_{2}=-k_{2}F+b_{2},$$

where F is the load, $k_{2}$ and $b_{2}$ represent the slope and intercept, respectively, and $k_{2}\;$> 0.

It can be inferred from Equation (3) that when the SVA of the LUSM remains constant, increasing the load of the LUSM will cause the speed to drop. Meanwhile, it can be inferred from Equation (2) that when the SVA of the LUSM increases, the speed of the motor will also increase. Therefore, by establishing the relationship between the load, SVA, and speed of the LUSM, the speed fluctuation caused by the load change can be compensated by the change of SVA.

### 2.2. Principle of the Stator Vibration Amplitude-Based Speed Control and the Frequency Tracking

**Figure 2:**
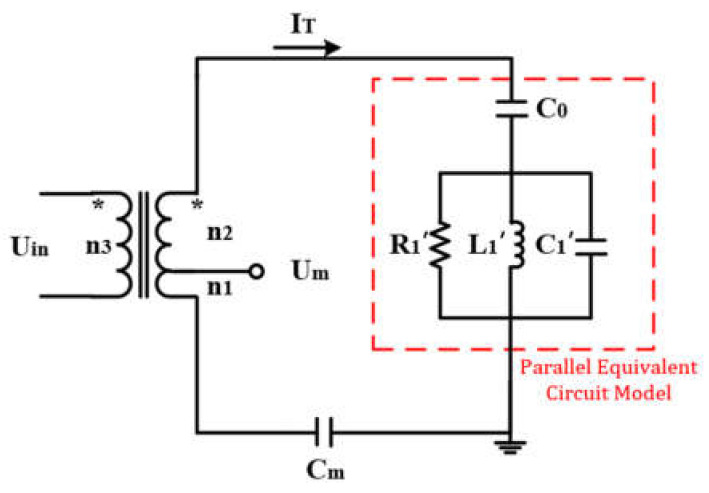
PZT ceramic parallel equivalent circuit and TRAB.

The working principles of LUSMs and rotary ultrasonic motors are similar, so in order to achieve the VBSC, a transformer ratio arm bridge (TRAB) is used to detect the SVA [19]. The parallel equivalent circuit model of the single-phase PZT ceramic of the LUSM and the schematic diagram of the TRAB are shown in Figure 2. $C_{0}$ represent the dielectric property, $R_{1}^{\prime }$, $L_{1}^{\prime }$ and $C_{1}^{\prime }$ can be expressed by the following equation:

(4) $$\left\{\begin{array}{c} R_{1}^{\prime }=\frac{1}{{\left(2\pi f\right)}^{2}C_{0}^{2}R_{1}}\\ L_{1}^{\prime }=\frac{C_{0}C_{1}/\left(C_{0}+C_{1}\right)}{{\left(2\pi f\right)}^{2}C_{0}^{2}}\\ C_{1}^{\prime }={\left(2\pi f\right)}^{2}C_{0}^{2}L_{1} \end{array}\right.,$$

where $R_{1}$, $L_{1}$, and $C_{1}$ represent damping, mass, and stiffness, respectively, these parameters are related to the characteristics of the LUSM, and f is the operating frequency of the LUSM, which is also the frequency of the drive signal. In addition, $C_{m}$ is the TRAB matching capacitance, $U_{in}$ is the input voltage, and $n_{1}$, $n_{2}$ and $n_{3}$ are the turns of the three-winding transformer. The value of $C_{m}$ needs to be set according to $n_{1}$, $n_{2}$ and $C_{0}$. When $n_{2}$ is much greater than $n_{1}$, the transformer tap voltage $U_{m}$ has a proportional relationship with SVA A, so the relationship between $U_{m}$ and A is [19]:

(5) $$A\propto kU_{m},$$

where k is the proportionality coefficient. It can be inferred from Equation (5) that the SVA linearly increases with the increasing of $U_{m}$, and because the SVA is linear with the speed of the LUSM, the VBSC can be achieved by changing the value of $U_{m}$. In the following, $U_{m}$ is used to represent the SVA.

In addition, temperature changes of the LUSM will also cause fluctuations in speed, reducing the stability of the LUSM [23,24], and changes in operating conditions will cause changes in the parallel resonance frequency ($f_{p}$). To improve the operating stability and efficiency, the operating frequency of the LUSM should be consistent with the $f_{p}$ [25,26], thus the frequency tracking of the LUSM is necessary. When the phase difference between $U_{m}$ and the input current $I_{T}$ is 0°, the LUSM operates at $f_{p}$ [19].

## 3. Specific Implementation Scheme

### 3.1. Calculation of the Stator Vibration Amplitude to Be Compensated

**Figure 3:**
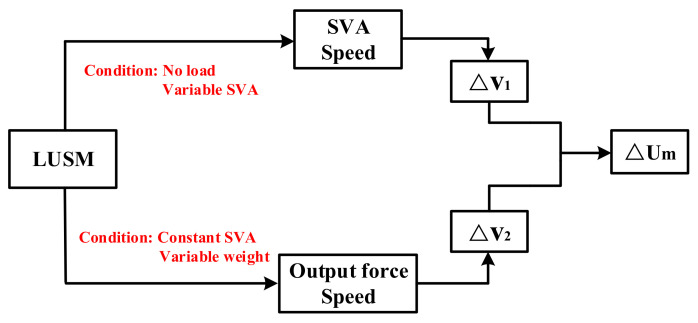
Calculation process of the stator vibration amplitude to be compensated.

The calculation process of the SVA to be compensated is shown in Figure 3. During the operation of the LUSM, frequency tracking is applied. First change the value of $U_{m}$ and set the LUSM to run at no load, use the laser gauging sensor (LGS) to record the speed of the LUSM under different SVAs, and use Equation (2) to fit the measurement results to get the stator vibration amplitude-speed curve. Then keep $U_{m}$ constant, change the output force of the LUSM by changing the weight, use the LGS to record the speed of LUSM under different output forces, and use Equation (3) to fit the measurement results to get the output force-speed curve.

Assuming the change of SVA is $\Delta U_{m}$, according to Equation (2), the velocity $v_{1}^{\prime }$ after the SVA change is:

(6) $$v_{1}^{\prime }=k_{1}\left(U_{m}+\Delta U_{m}\right)+b_{1}.$$

Then, the speed change $\Delta v_{1}$ is:

(7) $$\Delta v_{1}=v_{1}^{\prime }-v_{1}=k_{1}\Delta U_{m}.$$

In the same way, assuming the output force change is ΔF, according to Equation (3), the speed $v_{2}^{\prime }$ after the output force change is:

(8) $$v_{2}^{\prime }=-k_{2}\left(F+\Delta F\right)+b_{2}.$$

Then, the speed change $\Delta v_{2}$ is:

(9) $$\Delta v_{2}=v_{2}^{\prime }-v_{2}=-k_{2}\Delta F.$$

According to the above analysis, the speed fluctuation caused by the load change can be compensated by the change of the SVA, so let:

(10) $$\Delta v_{1}=\;-\Delta v_{2}.$$

Then we can derive:

(11) $$\Delta U_{m}=\frac{k_{2}}{k_{1}}\Delta F.$$

It can be deduced from Equation (11) that in order to eliminate the influence of load changes on the speed of LUSM, when the output force changes ΔF, $U_{m}$ needs to be compensated $\frac{k_{2}}{k_{1}}\Delta F$ on the basis of no load.

### 3.2. Hardware and Control Architecture

**Figure 4:**
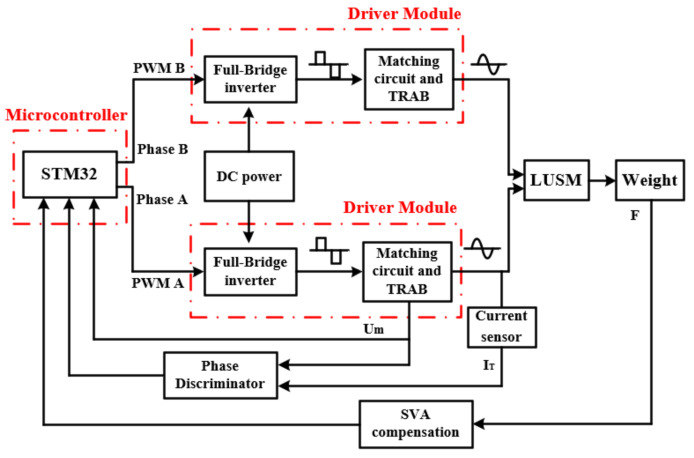
Hardware architecture of the proposed scheme.

The hardware architecture of the proposed scheme is shown in Figure 4. To generate two-phase drive signals with a phase difference of 90°, STM32 is used as a microcontroller to send two-phase pulse width modulation (PWM) signals with a phase difference of 90° to the driver module. The frequency and duty cycle of the PWM signals are adjusted according to the control requirements. The driver module is composed of a full-bridge inverter circuit, a matching circuit and a TRAB, in the proposed scheme, the ratio of turns $n_{1}$, $n_{2}$ and $n_{3}$ of the three-winding transformer is 1:10:1. The full-bridge inverter circuit converts the DC power into an AC square wave signal under the control of the PWM signal. The matching circuit is composed of an inductor, and the AC square wave signal becomes a sine wave signal after being filtered by the matching circuit. The sine wave signal is the drive signal of the LUSM. The frequency of the drive signal is equal to the frequency of the PWM signal. The voltage amplitude of the drive signal can be changed by adjusting the duty cycle of the PWM signal. The TRAB is used to further increase the voltage of the sine wave signal and detect the SVA. The output force F of the LUSM is changed by applying different weights and after SVA compensation, the target SVA is obtained and sent back to the microcontroller. In addition, to realize the VBSC and the frequency tracking, the microcontroller needs to measure the $U_{m}$ and the phase difference θ between $U_{m}$ and $I_{T}$. The microcontroller obtains the $U_{m}$ by measuring the transformer tap voltage and obtains the phase difference between $U_{m}$ and $I_{T}$ through the phase discriminator. Due to the symmetry of the stator structure, only single-phase signal is sampled.

**Figure 5:**
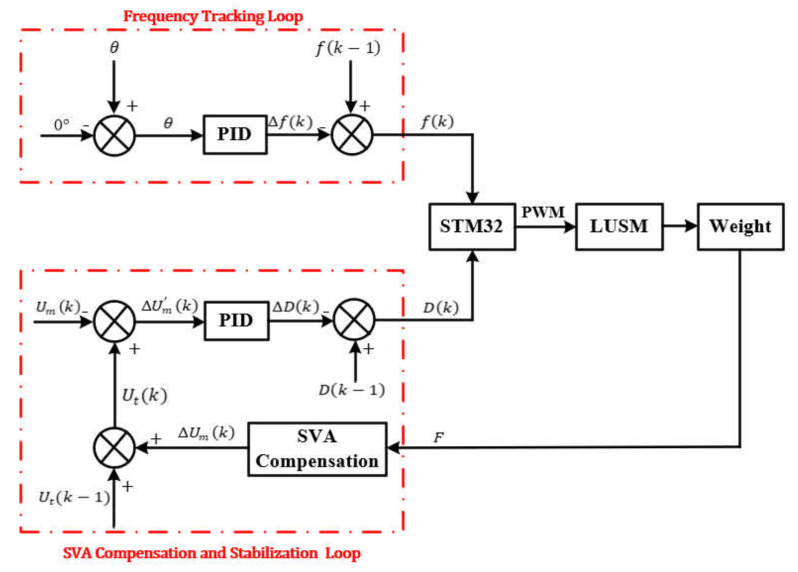
Control architecture of the proposed scheme.

The microcontroller adjusts the frequency and duty cycle of the PWM signal according to the sampled signal to achieve the purpose of changing the operating frequency and SVA of the LUSM. The control architecture of the proposed scheme is shown in Figure 5. The control architecture is mainly composed of the frequency tracking loop and the SVA compensation and stabilization loop. Because the sampling interval of $U_{m}$ and θ is slightly shorter than 25 us, the control period is set to 25 us.

To improve the operating efficiency and stability, the LUSM needs to operate at $f_{p}$. The operating frequency of the LUSM is equal to the frequency of the PWM signal, therefore, frequency tracking can be achieved by adjusting the frequency of the PWM signal. After the microcontroller receives the phase difference θ, incremental PID [27] is used to adjust the frequency $f\left(k\right)$ of the PWM signal until θ is equal to 0°. $f\left(k\right)$ and $\Delta f\left(k\right)$ are calculated as follows:

(12) $$\Delta f\left(k\right)=P_{f}\left[\theta \left(k\right)-\theta \left(k-1\right)\right]+I_{f}\theta \left(k\right)+D_{f}\left[\theta \left(k\right)-2\theta \left(k-1\right)+\theta \left(k-2\right)\right],$$

(13) $$f\left(k\right)=f\left(k-1\right)+\Delta f\left(k\right),$$

where $P_{f}$, $I_{f}$ and $D_{f}$ are the proportional, integral, and differential coefficients, respectively. In this experiment, the values of $P_{f}$, $I_{f}$ and $D_{f}$ are set to 0.5, 0.3, and 0.2. When the phase of $U_{m}$ leads the phase of $I_{T}$, i.e., θ > 0, the frequency of the PWM signal needs to be increased to reduce θ. Using the Equation (12), the PID controller output $\Delta f\left(k\right)$ is calculated according to the θ, and the frequency of the PWM wave $f\left(k\right)$ can be obtained from Equation (13). Conversely, when the phase of $U_{m}$ lags the phase of $I_{T}$, the frequency of the PWM signal needs to be decreased.

In the SVA compensation and stabilization loop, the speed is controlled by adjusting $U_{m}$. The microcontroller calculates the SVA to be compensated $\Delta U_{m}\left(k\right)$ by Equation (11) according to the change of the output force ΔF of the LUSM firstly. Then the target SVA $U_{t}\left(k\right)$ after compensation can be calculated by the following equation:

(14) $$U_{t}\left(k\right)=U_{t}\left(k-1\right)+\Delta U_{m}\left(k\right).$$

Set $U_{t}\left(k\right)$ as the target SVA, The current SVA $U_{m}\left(k\right)$ can be changed by adjusting the duty cycle of the PWM signal, the microcontroller calculates the error $\Delta U_{m}^{\prime }\left(k\right)$ between the $U_{m}\left(k\right)$ and the $U_{t}\left(k\right)$, and incremental PID is used to adjust the duty cycle $D\left(k\right)$ until $U_{m}\left(k\right)$ is equal to $U_{t}\left(k\right)$. $D\left(k\right)$ and $\Delta D\left(k\right)$ are calculated as follows:

(15) $$\Delta D\left(k\right)=P_{D}\left[\Delta U_{m}^{\prime }\left(k\right)-\Delta U_{m}^{\prime }\left(k-1\right)\right]+I_{D}\Delta U_{m}^{\prime }\left(k\right)+D_{D}\left[\Delta U_{m}^{\prime }\left(k\right)-2\Delta U_{m}^{\prime }\left(k-1\right)\;+\Delta U_{m}^{\prime }\left(k-2\right)\right],$$

(16) $$D\left(k\right)=D\left(k-1\right)+\Delta D\left(k\right),$$

where $P_{D}$, $I_{D}$ and $D_{D}$ are the proportional, integral, and differential coefficients, respectively. In this experiment, the values of $P_{D}$, $I_{D}$ and $D_{D}$ are set to 0.03, 0.003, and 0.002. When the current SVA $U_{m}\left(k\right)$ is smaller than the target SVA $U_{t}\left(k\right)$, i.e., $\Delta U_{m}^{\prime }\left(k\right)$ > 0, the duty cycle $D\left(k\right)$ needs to be increased to reduce $\Delta U_{m}^{\prime }\left(k\right)$. Using Equation (15), the PID controller output ∆D(k) is calculated according to the $\Delta U_{m}^{\prime }\left(k\right)$, and the $D\left(k\right)$ can be obtained from Equation (16). Conversely, when the $U_{m}\left(k\right)$ is greater than $U_{t}\left(k\right)$, the $D\left(k\right)$ needs to be decreased. 

## 4. Experimental Results and Discussion

### 4.1. Experimental Settings

**Figure 6:**
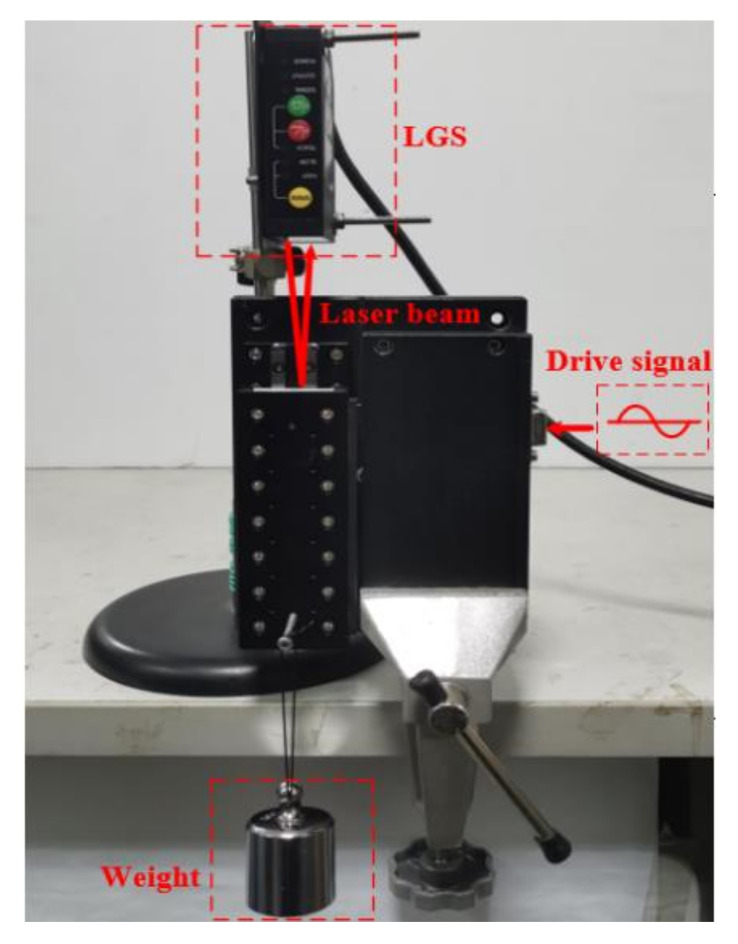
Experimental platform.

The experimental platform is shown in Figure 6. The 60Lumv LUSM developed by Nanjing University of Aeronautics and Astronautics is used to verify the feasibility of the proposed scheme. The motor is a V-shaped LUSM with a speed range of 80–1200 mm/s. The speed of the LUSM is measured by the banner laser gauging sensor (LGS) LG10A65PU. The LG10A65PU emits a laser beam to the slider, which is reflected by the slider surface and then transmitted back to the sensor. Thus, the position change of the slider can be measured. Then the speed of the LUSM can be calculated according to the position change and the time spent for the position change. Besides, for the easiness to analysis, the weight of the slider is ignored in the experiment.

### 4.2. Stator Vibration Amplitude Control and Frequency Tracking Verification

**Figure 7:**
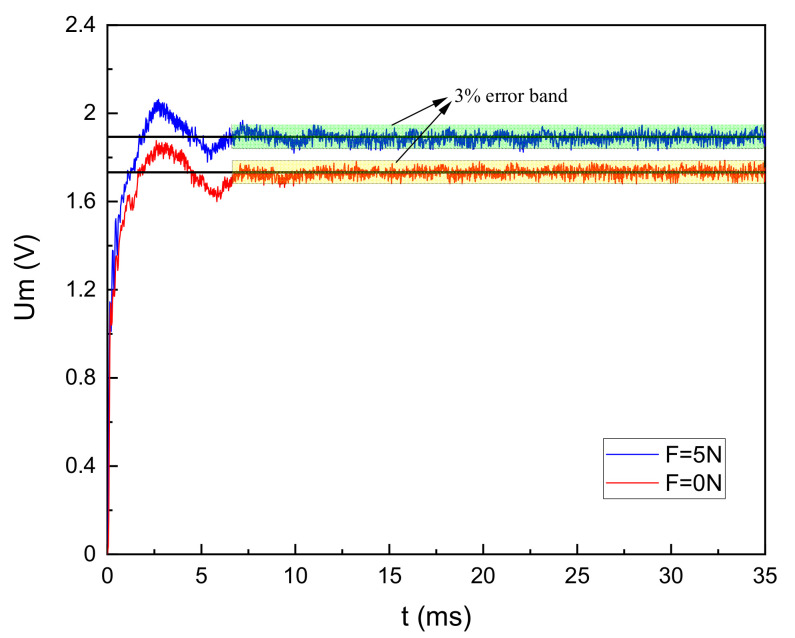
Stator vibration amplitude control process.

The goal of VBSC is to control $U_{m}$ to stabilize at the target SVA. Figure 7 shows the process of SVA control under no load and with a load. Set the target value of $U_{m}$ to 1.73V at no load. After the LUSM is equipped with a 500g weight, i.e., which means the output force is 5N, the target value of $U_{m}$ is set to 1.89V. In both cases, the fluctuation range of $U_{m}$ is about 3% after the LUSM is turned on for about 7ms, which verifies the feasibility of the SVA stabilization loop.

**Figure 8:**
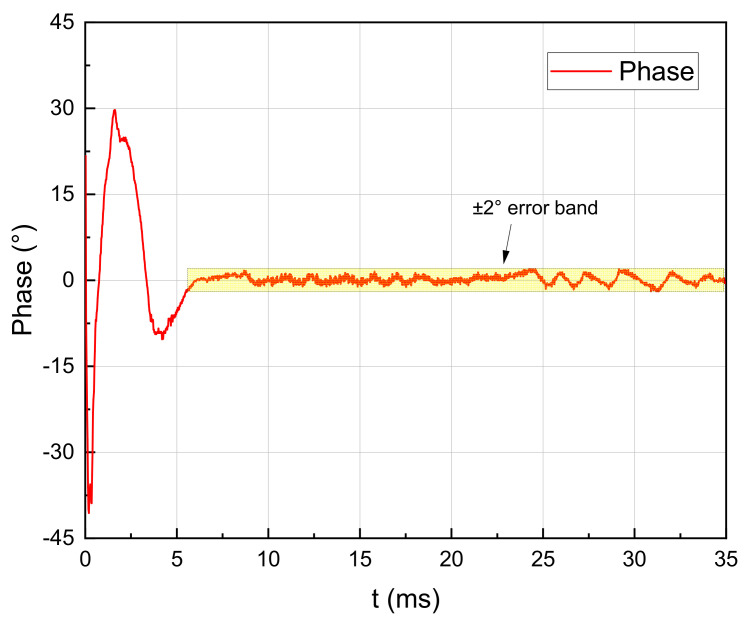
Frequency tracking process.

Figure 8 shows the frequency tracking process of the LUSM. After the LUSM is started, since the starting frequency is not the $f_{p}$, there is a certain phase difference between $U_{m}$ and $I_{T}$. The phase difference gradually decreases and finally remains around 0°, and the phase error is within ±2° in the stable state, which verifies that the frequency tracking makes the LUSM operate at the parallel resonance frequency.

### 4.3. Stator Vibration Amplitude-Speed, Output Force-Speed Curve and Calculation of the Stator Vibration Amplitude to Be Compensated

**Figure 9:**
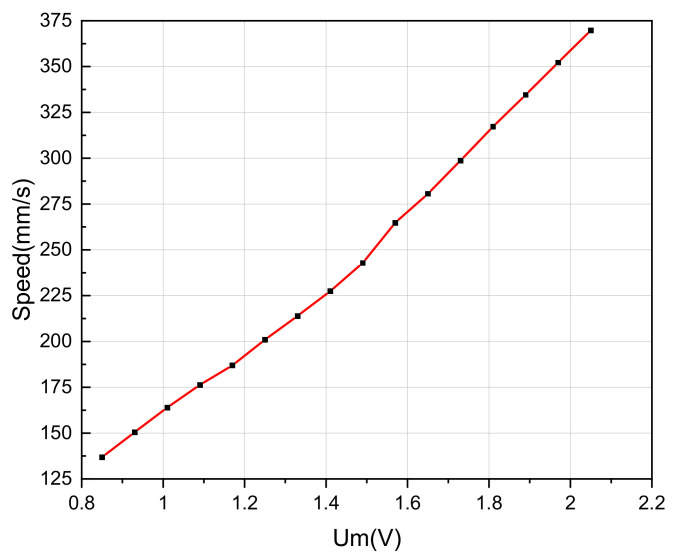
Relationship between stator vibration amplitude and speed.

On the basis of frequency tracking, the SVA control of the LUSM is carried out. Under no load conditions, measure the speed under different loads. According to the measurement result, when $U_{m}$ is less than 0.85V, the LUSM will become unstable, The reason is that when the LUSM reaches a lower speed, the drop of SVA will intensify the influence of the stick-slip phenomenon, which brings instability of movement [28]. Therefore, control $U_{m}$ to stabilize at 0.85V, 0.93V, 1.01V, 1.09V, 1.17V, 1.25V, 1.33V, 1.41V, 1.49V, 1.57V, 1.65V, 1.73V, 1.81V, 1.89V, 1.97V and 2.05V, respectively. Record the speed under different $U_{m}$. Repeat the measurement three times for each group of data and take the average value as the measurement results in improving the measurement accuracy. The relationship between the SVA and speed is shown in Figure 9. When the $U_{m}$ varies from 0.85-2.05V, it has a linear relationship with the speed, which is consistent with the theoretical analysis. It proves the SVA detection based on the TRAB is also suitable for LUSM.

**Figure 10:**
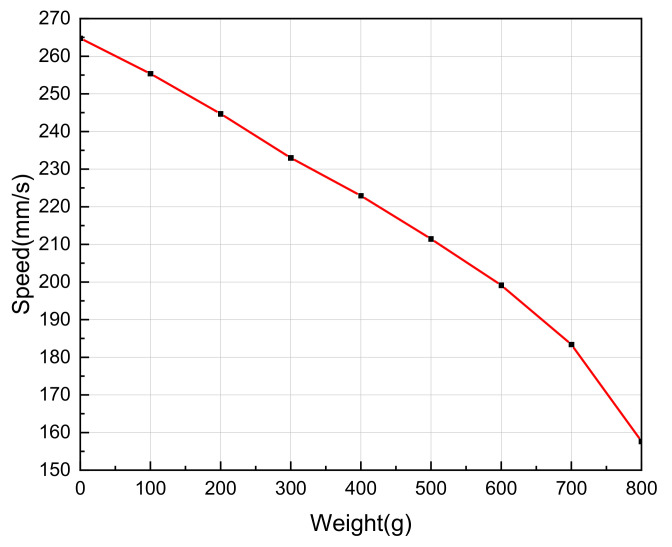
Relationship between weight and speed.

On the basis of frequency tracking, the $U_{m}$ is controlled to be constant at 1.57V, and the LUSM is equipped with weights. The weights are 100 g, 200 g, 300 g, 400 g, 500 g, 600 g, 700 g and 800 g, respectively. Record the speed under different output forces. Repeat the measurement three times for each group of data and take the average value as the measurement results in improving the measurement accuracy. The relationship between the output force and speed is shown in Figure 10. When the weight varies from 600–800 g, the relationship between the speed of the LUSM and output force is not linear. The reason is that when the load is greater than 600 g, the speed drops faster. However, when the weight is less than 600 g, as the output force increases, the speed of the LUSM decreases approximately linearly, which is consistent with the analysis in Section 2.1. To verify the feasibility of the proposed scheme, the case where the weight changes within 0–600 g are discussed. Meanwhile, the experimental results show that under the condition of constant SVA, as the output force increases, the speed of the LUSM fluctuates greatly. When the weight is added to 600 g, the speed decreases by 24.8% compared with the no load condition, i.e., when the load changes, the relationship between the SVA and speed is no longer linear.

**Figure 11:**
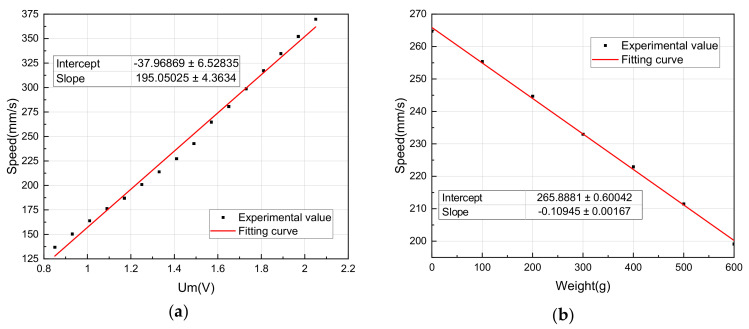
(**a**) Stator vibration amplitude-speed and (**b**) output force-speed curves.

Linear fitting is performed on the above measurement data, and the fitting result is shown in Figure 11.

According to Equation (2), the relationship between $U_{m}$ and the speed can be expressed by the following equation:

(17) $$v_{1}=k_{1}U_{m}+b_{1}=195.05025*U_{m}-37.96869$$

According to Equation (3), when $U_{m}$ = 1.57, the relationship between weight G and the speed can be expressed by the following equation:

(18) $$v_{2}=-k_{2}G+b_{2}=-0.10945*G+265.8881$$

According to Equation (11), it is obtained that whenever the weight increases by 100g, $U_{m}$ needs to be compensated:

(19) $$\Delta U_{m}=\frac{k_{2}}{k_{1}}\Delta G\;=\frac{0.10945}{195.05025}*100\approx 0.056V$$

To keep the speed of the LUSM under load consistent with the no load, $U_{m}$ needs to increase by 0.056V on the basis of no load whenever the output force increases by 1N.

### 4.4. Relationship between the Load and Speed After Stator Vibration Amplitude Compensation

**Figure 12:**
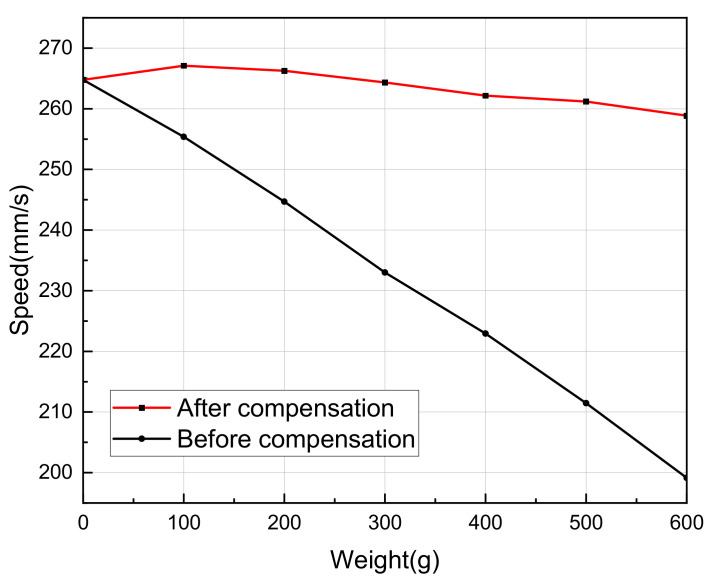
Speed of the linear ultrasonic motor under different loads

To verify the feasibility of the SSCBVC, the no load speed when $U_{m}$ is 1.57 V is set as the target of speed control. According to Equation (11), calculate the SVA to be compensated under different loads. The weights and the SVAs after compensation are shown in Table 1:

After SVA compensation, measure the speed under different loads. Repeat the measurement three times for each group of data and take the average value as the measurement result. The result is shown in Figure 12.

After the SVA compensation, as the weight increases, the speed fluctuation of the LUSM is significantly reduced compared to the condition without compensation. When the weight is 600 g, the error is about 2.2% compared with the speed under no load, which is much smaller than the error of 24.8% under the condition of constant SVA. The experiment shows that the SSCBVC can effectively reduce the influence of load changes on the speed of LUSM without using speed/position sensors.

However, as the load increases, the speed error tends to increase. One of the reasons is that under the condition of constant SVA, the relationship between the output force and speed is not completely linear. As the load increases, the speed of the LUSM decreases faster. This scheme simplifies the relationship between the load and speed of the LUSM and has the advantage of simple algorithm, but it is inevitable to introduce errors. If the speed control accuracy needs to be improved, the value of the SVA to be compensated should be further optimized. In addition, this scheme is suitable for cases where the load is less than a certain value. When the load exceeds this certain value, the SVA to be compensated also needs to be further optimized.

## 5. Conclusions

This paper proposes the SSCBVC of the LUSM to eliminate the influence of load changes on speed. According to the stator vibration amplitude-speed and the output force-speed curves of the LUSM, the relationship between the load changes and SVA be compensated is established. The load change will cause the speed fluctuation of the LUSM, and the SVA compensation can effectively offset the speed fluctuation caused by the load change. Compared with control methods that require speed/position sensors, the SSCBVC does not require these sensors and it can reduce the size and cost of the system; compared with neural network-based control methods, the SSCBVC does not require complex algorithms. The results show that the speed fluctuation is only 2.2% when the load increases by 6N. The proposed scheme can realize speed sensorless control of the LUSM under variable load conditions.

## Figures and Tables

**Table 1:** Weights and SVAs after compensation.

Weight (g)	SVA after Compensation (V)
0	1.57
100	1.626
200	1.682
300	1.738
400	1.794
500	1.85
600	1.906
